# Establishment of tongue microbiota by 18 months of age and determinants of its microbial profile

**DOI:** 10.1128/mbio.01337-23

**Published:** 2023-10-11

**Authors:** Shinya Kageyama, Jiale Ma, Michiko Furuta, Toru Takeshita, Mikari Asakawa, Yuka Okabe, Yoshihisa Yamashita

**Affiliations:** 1 Section of Preventive and Public Health Dentistry, Division of Oral Health, Growth and Development, Faculty of Dental Science, Kyushu University, Fukuoka, Japan; 2 OBT Research Center, Faculty of Dental Science, Kyushu University, Fukuoka, Japan; University of Maryland School of Medicine, Baltimore, Maryland, USA; University of Puerto Rico, Medical Sciences Campus, San Juan, Puerto Rico

**Keywords:** oral microbiota, 16S RNA, long-read sequencing, maturation, dental caries, dietary habit, breast milk

## Abstract

Acquisition of oral bacteria early in life is considered to have an important role in the establishment of oral microbiota. In this study, we examined tongue swab samples collected from 216 infants at the 18-month checkup, along with samples collected from them and from their mothers at the 4-month checkup. The bacterial composition of each sample was determined using PacBio single-molecule long-read sequencing of the full-length 16S rRNA gene and amplicon sequence variant (ASV) analysis. At 18 months of age, the tongue microbiota was dominated by *Streptococcus salivarius*, *Neisseria perflava*, and *Granulicatella adiacens*; and the overall bacterial composition was more similar to that of the mothers than at 4 months of age. The bacterial composition at 18 months of age was classified into three microbiota profiles: *S. salivarius*-dominant, *Neisseria*-dominant, and infant profile dominated by 4 months specific ASVs such as *S. salivarius*, *Streptococcus lactarius*, and *Streptococcus peroris*. Those with infant profiles exhibited significantly higher rates of current breastfeeding, dental caries or white spot lesions, and dental plaque accumulation compared to infants with the other profiles. Comparison between the *S. salivarius*- and *Neisseria*-dominant profiles showed that incomplete weaning, low intake of fruits, and frequent intake of sweetened beverages or sweet snacks were significantly associated with the *S. salivarius*-dominant profile. Our findings, based on a high-resolution profiling approach, demonstrate that the foundation of the adult tongue microbiota is established by 18 months of age, and the bacterial profile is possibly determined by dietary habits during this period.

**IMPORTANCE:**

Understanding the development of oral microbiota early in life and the factors that influence it is important for preventing the establishment of dysbiotic oral microbiota later in life. This study demonstrates that the tongue microbiota undergoes early development from 4 to 18 months of age and converges into two types of microbiota showing indications of adult characteristics, with either *S. salivarius* or *Neisseria*-dominance. Interestingly, their divergence was strongly determined by their weaning status and the dietary frequencies of sweetened beverages, snacks, and fruits, suggesting that dietary habits during this period might influence the establishment of the oral microbiota. These findings may contribute to the development of novel preventive strategies against oral microbiota-related diseases.

## INTRODUCTION

The human body hosts numerous microbes, which form unique microbial ecosystems in different body sites (1, 2). The gastrointestinal tract is a major microbial reservoir, and the intestinal microbiota plays an essential role in human health and physiology (3, 4). The oral cavity also harbors a large microbial community, and the oral microbiota is well known to be associated with oral diseases such as dental caries and periodontitis (5 – 7). Interestingly, the oral microbiota has recently attracted attention as a microbial source for the digestive tract and respiratory system, and its relevance to diseases of these distant organs has been suggested (8 – 10). Several studies have demonstrated an abnormal enrichment of oral bacteria in the gut microbiota of patients with liver cirrhosis, inflammatory bowel diseases, and colorectal cancer (11 – 13). Although the causal role of oral microbiota composition in these diseases is poorly understood, elucidating how oral microbiota is established and maintained might contribute to the development of novel preventive and treatment strategies for oral microbiota-related diseases.

At delivery, the neonatal oral microbial community is relatively homogeneous with the gut, nasal, and skin communities; however, at 6 weeks of age, the formation of an indigenous oral microbiota begins (14). The oral microbiota at this stage shows a completely different bacterial composition compared with the maternal oral microbiota, and this marked difference was also observed at 4 months of age (14, 15). Subsequently, the oral microbiota has been reported to diversify and approach the adult composition through alterations in the oral environment derived from the eruption of teeth or the start of solid food ingestion at around 2 years of age (16 – 18). Interestingly, some bacteria that colonize the oral cavity during early infancy remain there until adulthood as consistent core bacteria (18, 19). For instance, a longitudinal study verified that *Streptococcus salivarius*, *Streptococcus mitis* group species, and *Streptococcus sanguinis*, which are commonly observed in the adult oral microbiota, colonized the oral cavity by 1 or 2 years of age and continuously dominated the oral microbiota until 7 years of age (19). Given their persistent colonization, the early oral microbiota formed by 2 years of age may lay the foundation for the subsequent colonization of oral bacteria and the formation of oral microbiota in adulthood. Further, the identification of influential factors in the establishment of this early oral microbiota might enable the healthy development of adult oral microbiota.

In this study, we examined 216 tongue swab samples collected from infants at 18 months of age and compared them to maternal and personal samples collected at 4-month checkup. The bacterial composition was determined using full-length 16S rRNA gene sequences and the amplicon sequence variant (ASV) approach (20, 21). The combination of these approaches enables the enhancement of taxonomic discrimination and the determination of bacterial composition at high resolution (15, 22). This study aimed to precisely profile the infant microbiota at 18 months of age using these approaches, and to identify the relevant factors influencing microbial profiles.

## RESULTS

### Study population and full-length 16S rRNA gene sequences

Of the 448 infants who participated in our previous study at their 4-month checkup (15), we followed up and examined 216 infants (105 boys and 111 girls) during a dental checkup at 18 months of age. The detailed characteristics of the 216 infants are presented in Table S1. At the 18 months dental checkup, 66.7% of the infants had been weaned, whereas 33.3% were still breast- or formula-fed (19.4% were exclusively breastfed, 10.6% were exclusively formula-fed, and 3.2% were fed with both). Dental caries or white spot lesion were detected in only three infants (1.4%), and dental plaque accumulation was observed in only seven infants (3.2%). We collected tongue swab samples and determined their bacterial composition by full-length 16S rRNA gene amplicon analysis using PacBio long-read sequencing. In this study, we included in the analyses samples from the infants and from their mothers collected at the 4-month checkup. Briefly, 645 samples (216 triads, including three mothers of twins) were analyzed in this study. After denoising by DADA2 (21), 3,283,945 denoised reads [5,716.0 ± 1,665.1 reads (mean ± standard deviation) per infant sample at 4 months of age, 4,119.9 ± 1,608.3 per infant sample at 18 months of age, and 5,346.5 ± 2,362.4 per maternal sample] and 9,067 ASVs were obtained. Of all ASVs, 8,775, accounting for 99.6% of all reads, exhibited ≥98.5% identity with the reference sequences in eHOMD (23).

### Tongue microbiota composition at different ages

**Fig 1:**
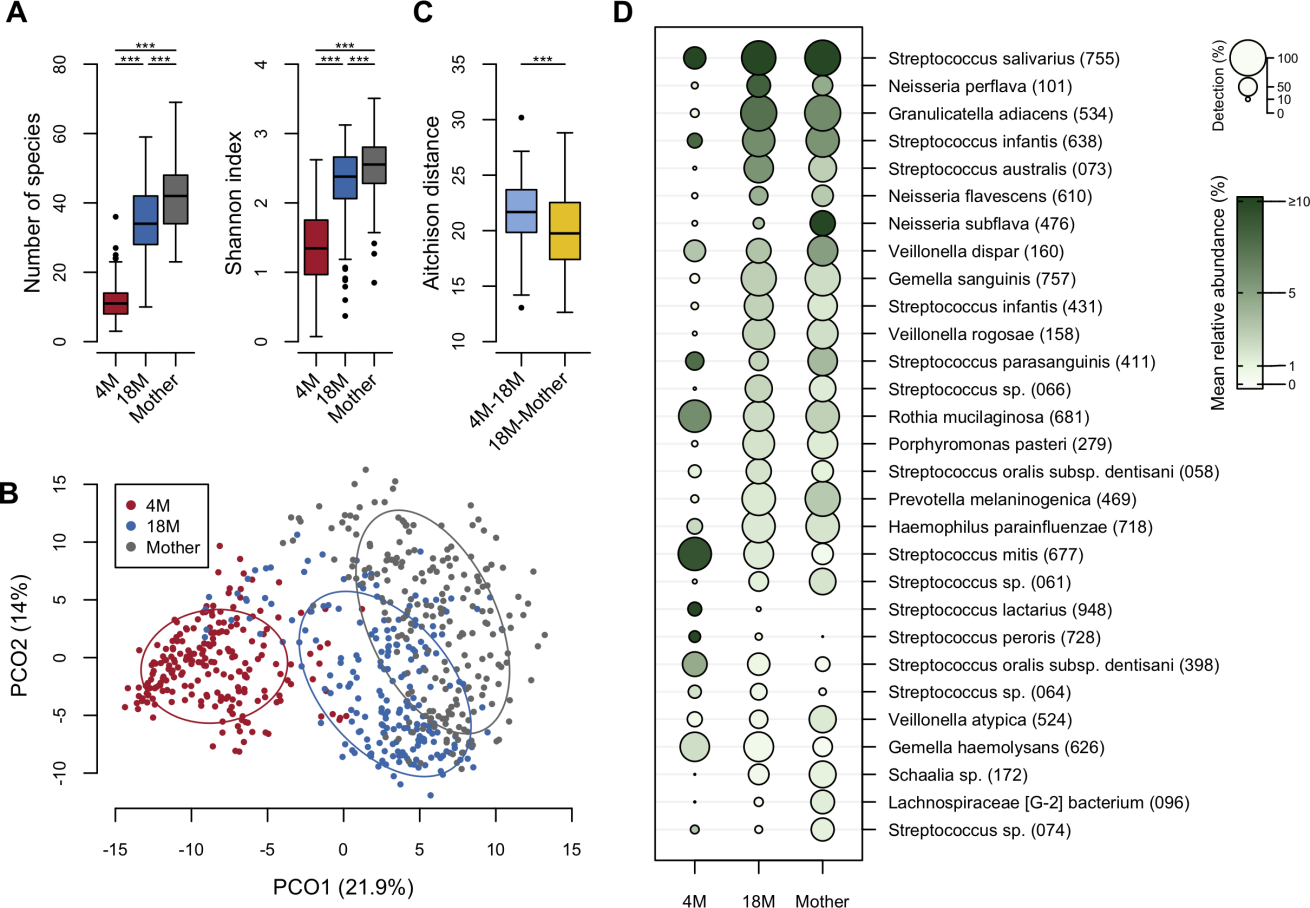
Compositional difference of tongue microbiota in the 4 months (4M), 18 months (18M), and maternal groups. (**A**) Number of species and Shannon index in each group. Significance was calculated using the Steel–Dwass test. ***P < 0.001. (**B**) Principal coordinate analysis plot of infant and maternal samples using Aitchison distance at species-level. The bacterial compositions of infant samples at 4 months and 18 months of age and of maternal samples are depicted using different colors. The ellipse covers 67% of the samples belonging to each group. (**C**) The Aitchison distance of 4 months–18 months pairs and of 18 months–mother pairs in each triad. The significance was calculated using the Wilcoxon signed-rank test. ***P < 0.001. (**D**) Relative abundance and detection ratio of predominant species. Twenty-nine species with a mean relative abundance ≥1% in either group are listed in descending order of mean relative abundance in infant microbiota at 18 months.

First, we compared the overall bacterial composition of tongue microbiota in infant samples collected at 4 and 18 months of age, and in maternal samples. Although the alpha diversity of the tongue microbiota at 18 months was still significantly lower than that of the maternal microbiota (Fig. 1A), the bacterial composition at 18 months overlapped more with the maternal composition than the composition at 4 months, according to the principal coordinate analysis plot based on the Aitchison distance (Fig. 1B). In fact, the distance between 18 months and mother pairs was significantly lower than that between 4 months and 18 months pairs (Fig. 1C). At 18 months of age, *S. salivarius* HOT-755, *Neisseria perflava* HMT-101, *Granulicatella adiacens* HMT-534, *Streptococcus infantis* HMT-638, and *Streptococcus australis* HMT-073 were predominant in the tongue microbiota (Fig. 1D). Notably, of 21 bacterial species with ≥1% of mean relative abundance at 18 months of age, 11 were also dominant at 4 months of age, whereas as many as 19 were dominant in maternal samples.

### Tongue microbiota profiles at 18 months of age

**Fig 2:**
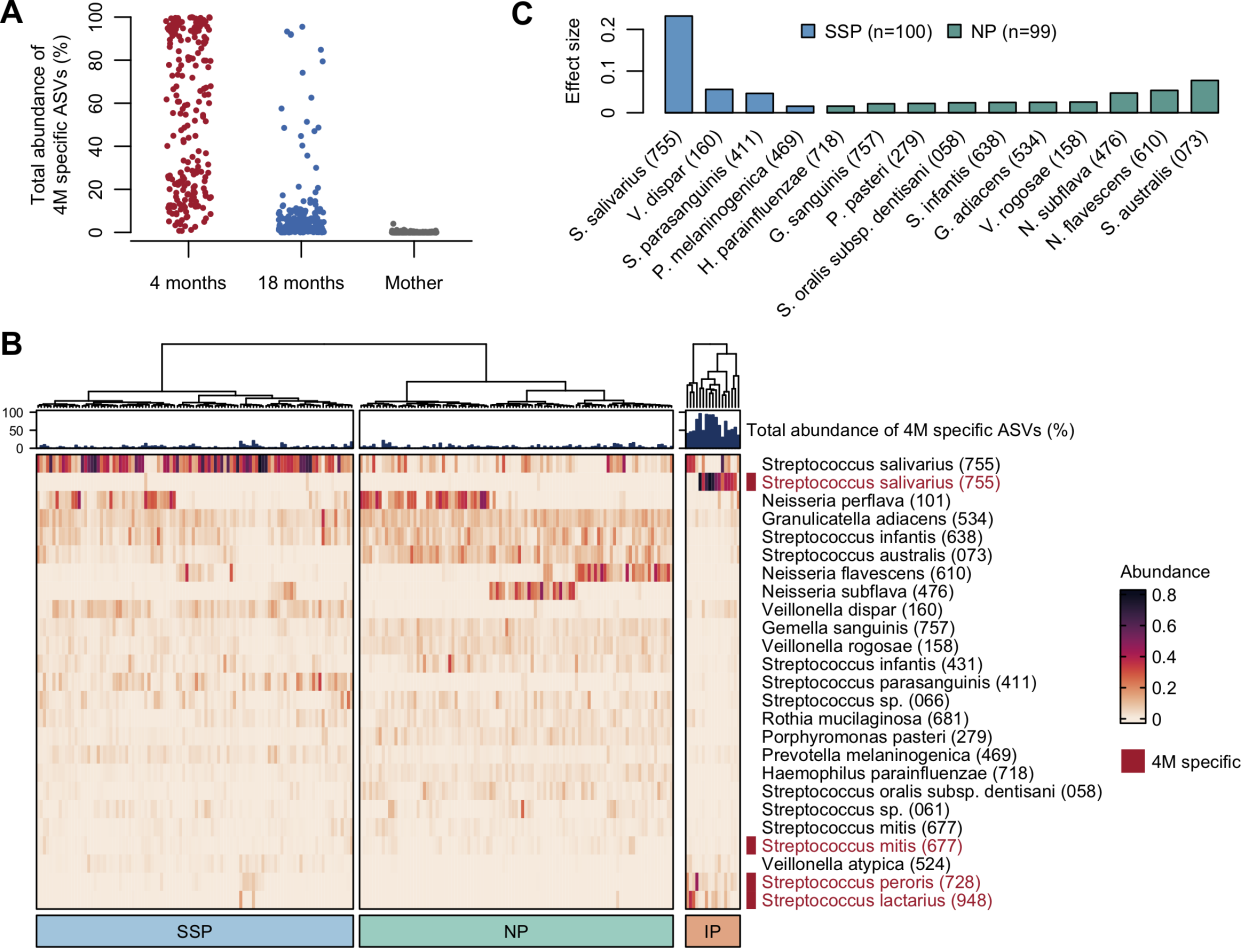
Tongue microbiota profiles at 18 months of age. (**A**) Total abundance of 4-month (4M) specific ASVs in each group. (**B**) Heatmap of bacterial composition of infant microbiota at 18 months of age. Species-level sub-units with a mean relative abundance ≥0.5% are shown, and sub-units composed of the 4M-specific ASVs are shown in red. The relative abundance of each unit is represented by color intensity. Each bar plot indicates the total abundance of 4M-specific ASVs in each microbiota. The hierarchical clustering in infant or mature profile (*S. salivarius*- and *Neisseria*-dominant profiles) is performed based on the Aitchison distance. IP, infant profile; NP, *Neisseria*-dominant profiles; SSP, *S. salivarius*-dominant profile. (**C**) The differentially abundant species between SSP and NP. Bar plots show the effect size of the MaAsLin 2 in each species. The species with a *q* value <0.05 and effect size ≥0.01 are shown.

Next, we profiled the bacterial composition of the tongue microbiota at 18 months of age. For profiling, including the consideration of microbiota maturity, we first identified and distinguished ASVs that were specific to infants at 4 months of age compared to mothers as an indicator of delayed maturation. These 4-month specific ASVs accounted for a median relative abundance of 54.6% in tongue microbiota at 4 months of age, whereas they accounted for only 3.4% and 0.0% in the tongue microbiota in infants at 18 months of age and in mothers, respectively (Fig. 2A). Based on the distribution of their relative abundance, we classified the tongue microbiota of infants at 18 months of age into infant (with ≥30%, *n* = 17) and mature profiles (<30%, *n* = 199) (Fig. S1). In the infant profile, 4-month specific ASVs corresponding to *S. salivarius*, *Streptococcus lactarius* HMT-948, and *Streptococcus peroris* HMT-728 were predominant (Fig. 2B). The mature profile was further subdivided into a *S. salivarius*-dominant profile (*n* = 100) and a *Neisseria*-dominant profile (*n* = 99) by hierarchical cluster analysis based on the Aitchison distance. In the *S. salivarius*-dominant profile, *S. salivarius*, *Veillonella dispar* HMT-160, and *Streptococcus parasanguinis* HMT-411 were differentially abundant (*q* < 0.05; Fig. 2C). *S. australis*, *Neisseria flavescens* HMT-610, and *Neisseria subflava* HMT-476 were differentially abundant in the *Neisseria*-dominant profile.

### Tongue microbiota profiles and infant characteristics

**Fig 3:**
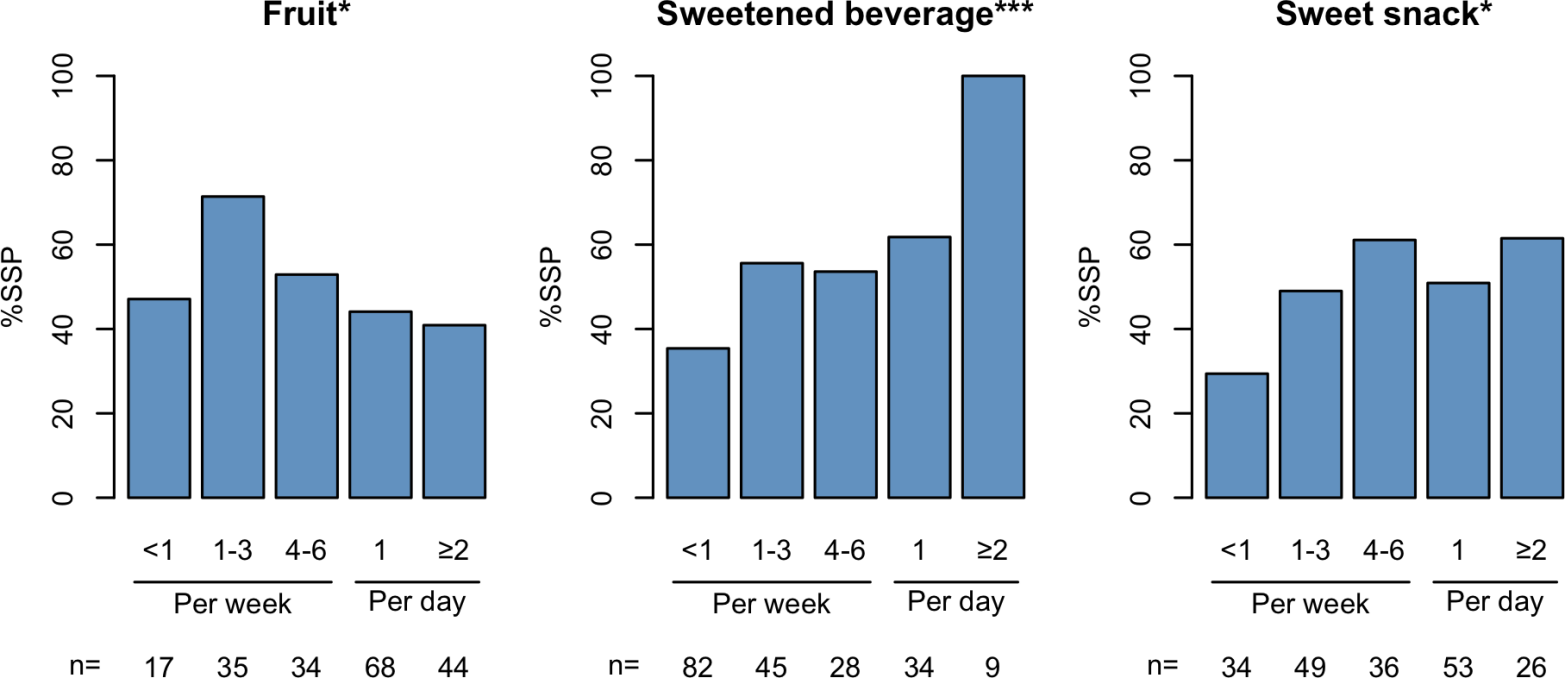
Linear trends in the proportion of the *S. salivarius*-dominant profile (SSP) across frequencies of dietary intake in infants with a mature profile. The number of participants in each category is shown below each bar plot. The significance was calculated using the Cochran-Armitage trend test. *P < 0.05, ***P < 0.001.

Finally, to identify the relevant factors influencing the tongue microbiota profiles of infants at 18 months of age, we compared the characteristics of infants with mature and infant tongue microbiota profiles, and the *S. salivarius*- and *Neisseria*-dominant profiles. Interestingly, the feeding method was strongly predictive of the presence of the mature or infant profile (*P* < 0.001, Table 1). In particular, all weaned infants demonstrated a mature profile, and most infants with infant profiles were being breastfed at the time (82.4%). In addition, dental caries, including white spot lesion, were detected only in infants with the infant profile, and dental plaque accumulation was significantly higher in these infants (both *P* < 0.001). Comparing infants with *S. salivarius*- and *Neisseria*-dominant profiles, incomplete weaning, a low intake of fruits, and frequent intake of sweetened beverages or sweet snacks were associated with the *S. salivarius*-dominant profile (Table 2). Multivariate analysis also demonstrated a significant association between a frequent intake of sweetened beverages and the *S. salivarius*-dominant profile (Table S2). Trend analysis demonstrated that the frequencies of sweetened beverage and sweet snack intake were significantly associated with increasing proportions of the *S. salivarius*-dominant profile, and the frequencies of fruit intake showed significant inverse associations with the proportions of this profile (Fig. 3). Furthermore, the *S. salivarius*-dominant profile was more frequent in infants who shared tableware with adults compared to those who did not share tableware (Table 2; Table S2).

**TABLE 1:** Characteristic of infants with mature and infant profiles at the 18-month checkup^*a*^

	Mature profile (*n* = 199)	Infant profile (*n* = 17)	*P* value
Age (months)	18.0 (17.6–18.5)	17.9 (17.7–18.0)	0.270
Boys	96 (48.2)	9 (52.9)	0.803
Feeding method			
Breastfed	28 (14.1)	14 (82.4)	<0.001
Mixed-fed	5 (2.5)	2 (11.8)	
Formula-fed	22 (11.1)	1 (5.9)	
Weaned	144 (72.4)	0 (0)	
Number of present teeth	16 (14–16)	16 (13–16)	0.662
Dental caries or white spot lesion	0 (0)	3 (17.6)	<0.001
Dental plaque accumulation	2 (1.0)	5 (29.4)	<0.001
Toothpaste with fluoride	121 (60.8)	9 (52.9)	0.608
Fluoride treatment at dental office	49 (24.7)	2 (11.8)	0.372
Brushing of teeth by mother	196 (98.5)	16 (94.1)	0.281
Tableware sharing with adults	95 (47.7)	10 (58.8)	0.453
Daycare center attendance	111 (55.8)	8 (47.1)	0.613
Antibiotic within a month	32 (16.2)	3 (17.6)	0.744
Dietary intake (≥4 times per week)			
Fruits	146 (73.7)	12 (70.6)	0.778
Dairy products	157 (79.7)	11 (64.7)	0.213
Sweetened beverages	71 (35.9)	6 (35.3)	1
Sweet snacks	115 (58.1)	14 (82.4)	0.069

^*a*^ Data are presented as median values (interquartile range) for age and number of teeth present, and *n* (%) for categorical variables.

**TABLE 2:** Characteristic of infants with *S. salivarius*- and *Neisseria*-dominant profiles at the 18-month checkup^*a*^

	*S. salivarius*-dominant profile (*n* = 100)	*Neisseria*-dominant profile (*n* = 99)	*P* value
Age (months)	18.0 (17.5–18.6)	18.0 (17.6–18.5)	0.499
Boys	52 (52.0)	44 (44.4)	0.322
Feeding method			
Breastfed	21 (21.0)	7 (7.1)	0.002
Mixed-fed	3 (3.0)	2 (2.0)	
Formula-fed	15 (15.0)	7 (7.1)	
Weaned	61 (61.0)	83 (83.8)	
Number of present teeth	16 (14–16)	16 (13.5–16)	0.327
Dental plaque accumulation	1 (1.0)	1 (1.1)	1
Toothpaste with fluoride	66 (66.0)	55 (55.6)	0.148
Fluoride treatment at dental office	22 (22.2)	27 (27.3)	0.510
Brushing of teeth by mother	98 (98.0)	98 (99.0)	1
Tableware sharing with adults	60 (60.0)	35 (35.4)	<0.001
Daycare center attendance	58 (58.0)	53 (53.5)	0.569
Antibiotic within a month	16 (16.2)	16 (16.2)	1
Dietary intake (≥4 times per week)			
Fruits	66 (66.7)	80 (80.8)	0.035
Dairy products	76 (77.6)	81 (81.8)	0.483
Sweetened beverages	45 (45.5)	26 (26.3)	0.007
Sweet snacks	65 (65.7)	50 (50.5)	0.043

^*a*^ Data are presented as median values (interquartile range) for age and number of teeth present and *n* (%) for categorical variables.

## DISCUSSION

This study examined 216 tongue swab samples collected from infants during their 18-month checkup, and profiled their tongue microbiota composition at high resolution using full-length 16S rRNA gene amplicon analysis. At only 18 months of age, the bacterial composition of the tongue was significantly more similar to that of their mothers compared to the one they exhibited at 4 months of age, and showed two distinct microbial profiles characterized by the predominance of either *S. salivarius* or *Neisseria*. These oral microbiota profiles were similar to those observed later in life in primary school children, adolescents, community-dwelling adults, and nursing home residents (7, 24 – 27). These findings suggest that the tongue microbiota develops between 4 and 18 months of age, and that the foundation of the adult tongue microbiota might be established during this period.

Tongue microbiota composition dominated by *S. salivarius*, *S. parasanguinis*, *Veillonella*, and *Prevotella* is reportedly associated with an increased risk of dental caries and pneumonia (25, 26). For instance, the total relative abundance of such bacterial groups in the tongue microbiota is positively correlated with the salivary levels of mutans streptococci, a known causal agent of dental caries (24, 25). The microbial composition of the *S. salivarius*-dominant profile observed in the present study was consistent with those that have been implicated in disease development (Fig. 2C). This suggests that the formation of such tongue microbiota begins within the first 18 months of life. Interestingly, the *S. salivarius*-dominant profile was predominant in infants with incomplete weaning, a low intake of fruits and frequent intake of sweetened beverages or sweet snacks (Table 2; Fig. 3). A systematic review reported that high sugar intake is associated with a predominance of *Streptococcus* and *Veillonella* in the oral cavity (28). This may be due to frequent acidification of the oral environment (27, 29). Contrarily, fruit intake was associated with the *Neisseria*-dominant profile rather than the *S. salivarius*-dominant profile, despite the high sugar content of fruits. The confounding factors, such as other dietary components, type differences in carbohydrates, and socioeconomic factors, could account for this observation. Further nutritional analyses focusing on the type and amount of fermentable carbohydrates or other dietary components are required to elucidate the relationship between a sugary diet and oral microbiota.

At the 18-month dental checkup, one-third of the infants (*n* = 72) were not completely weaned. These infants had a significantly higher ratio of the infant profile compared to weaned infants, and this ratio was particularly high in those who were being breastfed (Table 1). Interestingly, none of the weaned infants showed an infant profile. These results suggest that long-term breastfeeding delays the maturation of the oral microbiota. This is consistent with several studies reporting that breastfeeding is associated with lower microbial diversity and maturity in the oral and gut microbiota (15, 19, 30, 31). Breast milk provides protective factors, including immunoglobulins, leukocytes, lactoferrin, and lysozymes, and is thought to play a role as gatekeeper while the infant’s immune system is still immature (29, 32). Meanwhile, metabolic products of *Streptococcus* species on human milk oligosaccharides have been suggested to encourage the attachment and growth of selected oral commensal bacteria (33). Although the influence of breast milk components on the oral microbiota is still poorly understood, these conflicting functions are likely to play a role in the colonization of the oral cavity by bacteria, and maintain the infant tongue microbiota at a lower level of maturity.

Although dental caries or white spot lesions were only observed in three infants, the prevalence was significantly associated with breastfeeding at 18 months of age, dental plaque accumulation, and the infant profile of tongue microbiota (Table 1). All three infants were still being breastfed, had dental plaque accumulation, and showed the infant profile. A systematic review reported that breastfeeding beyond 12 months is associated with an increased risk of dental caries compared to breastfeeding for up to 12 months (34). Considering that dental plaque bacteria are directly responsible for dental caries, prolonged breastfeeding could contribute to cariogenesis through the accumulation of dental plaque due to the constant contact between breast milk (which contains lactose, a fermentable carbohydrate) and the tooth surface (35). Meanwhile, the infant profile was significantly associated with increased dental plaque (28.6%) compared to the mature profile (3.6%), even in the stratified analysis of breastfed infants. This result suggests that a less mature tongue microbiota may also be associated with dental plaque accumulation, independent of breastfeeding. However, the infant profile predominantly consists of *S. salivarius* (infant specific), *S. lactarius*, and *S. peroris*, and they normally disappear shortly after eruption of teeth, possibly through completion of weaning (18); thus, the influence of persistent colonization of such bacterial species on dental plaque formation and dental caries has not been investigated. Focusing on the relationships between prolonged breastfeeding, dental plaque accumulation, and the persistence of these infant-specific bacteria on the tongue dorsum may provide a novel perspective for understanding the etiology of dental caries.

Although a continuation of feeding at 18 months of age was strongly associated with tongue microbiota profiles, the feeding method at the 4-month checkup demonstrated no significant association with the profile at 18 months (Table S3). Weaning age may be important in the formation of tongue microbiota, regardless of feeding method. Notably, self-reported tableware sharing with adults was associated with a *S. salivarius*-dominant profile. This association was also observed in the multivariate analysis concerning dietary habits (Table S2). This suggests that tableware sharing with adults has an impact on the tongue microbiota profile, independently of dietary habits. Although frequent exposure to adult oral microbiota may play a role in the establishment of tongue microbiota during infancy, potential confounding factors cannot be excluded. Therefore, this association should be carefully considered in future studies.

The bacterial composition at 18 months of age was similar to that of the mother, while the 4-month specific ASVs were still broadly detected at that age (Fig. S2). Furthermore, the alpha diversity of the tongue microbiota at 18 months was lower than that of the maternal microbiota (Fig. 1A). These results indicate that the tongue microbiota at 18 months of age is still developing toward adult microbiota. Interestingly, 4-month specific ASVs were also detected in 29.1% of the maternal samples as a minor component (Fig. 2; Fig. S2). These were likely sourced from daily physical contact with infants at 4 months of age. Although it is unclear whether infant-derived bacteria persist in the maternal oral cavity, childcare may influence maternal oral microbiota.

This study had several limitations. First, to evaluate the effect of antibiotic use on infant oral microbiota, the study subjects included infants who were administered antibiotics within a month. Results similar to our present results were obtained after excluding antibiotic users from the analysis (Tables S4 and S5). Second, the follow-up rate was only 48.2% owing to the COVID-19 pandemic. However, there were no significant differences in the oral health conditions at the 4-month checkup between the included and excluded subjects (Table S6). The COVID-19 pandemic has also prevented us from obtaining general health information. Further studies are required to elucidate the relationship between tongue microbiota profiles at 4 and 18 months of age and general health conditions. Third, since this study was conducted as part of public health services, international standard criteria, such as the World Health Organization (WHO) or International Caries Detection and Assessment System (ICDAS) criteria for dental caries, were not used. Fourth, we examined only the tongue microbiota in this study; to study the relationship between the oral microbiota and dental caries in detail, the dental plaque microbiota should be examined in future studies.

In conclusion, full-length 16S rRNA gene amplicon analysis demonstrated that tongue microbiota undergoe early development from 4 to 18 months of age. During this period, the microbiota of most infants developed into two different profiles with signs of adult traits, *S. salivarius*- and *Neisseria*-dominant profiles, and their divergence was strongly determined by their weaning status and intake of sweetened beverages, snacks, and fruits. These findings suggest that dietary habits during this period are the key determinants of tongue microbiota profiles. The long-term impact of diet on the tongue microbiota should be further studied using a longitudinal approach.

## MATERIALS AND METHODS

### Study subjects and data collection

The subjects of this study were infants who attended a dental checkup at 18 months of age from March to June 2021. Of the 448 infants at 4 months of age who participated in our previous study, only 216 (48.2%) participated in this follow-up study. We conducted a dental examination and collected tongue swab samples by scraping the dorsum of the tongue with a Puritan Hydraflock swab (Puritan Medical Products, Guilford, ME, USA). Teeth with visible cavities were recorded as having dental caries according to the fifth edition of WHO oral health surveys: basic methods (36), and white spot lesions when wet (corresponding to ICDAS code 2) were also examined (37). Dental plaque was evaluated by examining the labial surface of the upper deciduous central and lateral incisors, and adhesion to over one-third of these surfaces was recorded as dental plaque accumulation. The collected samples were transported to our laboratory and stored at −80°C until further analysis, as previously described (15). Clinical information on the feeding method at the time, antibiotic use, fluoride application, daycare center attendance, tableware sharing with adults, brushing by adults, and dietary intake was obtained from our questionnaire. Written informed consent was obtained from all mothers at the 4-month checkup. The Ethics Committee of Kyushu University approved the present study and the procedure for obtaining informed consent (approval number: 2022-94).

### Full-length 16S rRNA gene amplicon sequencing

DNA was extracted from each sample using the bead-beating method, as previously described (15). The full-length 16S rRNA gene containing all variable regions was amplified using the following primers with the sample-specific 8-base tag sequence: 8F (5’-AGA GTT TGA TYM TGG CTC AG-3‘) and 1492R (5‘-GGY TAC CTT GTT ACG ACT T-3‘). PCR amplification and purification were performed as previously described (15). The purified amplicons were sequenced using the Sequel II Sequencing Kit 2.0 (Pacific Biosciences, Menlo Park, CA, USA) on a PacBio Sequel IIe (Pacific Biosciences) at the Kazusa Genome Technologies and circular consensus sequencing (CCS) reads were generated. From all the obtained CCS reads, the high-fidelity (HiFi) reads with ≥3 full-pass subreads and ≥20 quality values were finally used for analysis.

### Data analysis and taxonomy assignment

Data analysis was performed using HiFi reads from 216 infants at the 18-month checkup and those previously from them and their mothers at the 4-month checkup (216 and 213 samples, respectively) (15). The HiFi reads were primarily quality checked using R software (version 3.6.2, R Foundation for Statistical Computing, Vienna, Austria) and were excluded from the analysis when they exhibited <1,000 bases or did not include the correct forward and reverse primer sequences. The remaining HiFi reads were demultiplexed by examining the 8-base tag sequences at both ends, and the forward and reverse primer sequences were trimmed. The quality-checked HiFi reads were further processed using the DADA2 pipeline (version 1.14.0) with default settings for PacBio reads (except for the pseudo pooling) and an ASV table was produced (21). Seventeen ASVs observed in the negative control, predominantly *Pseudomonas fluorescens*, were excluded from the ASV table and subsequent analysis as PCR contaminants. Species-level taxonomic assignment for each sequence variant was performed using BLAST (38) against 1,015 oral bacterial 16S rRNA gene sequences (16S rRNA RefSeq version 15.22) in eHOMD (23). Nearest-neighbor species with ≥98.5% identity was selected as candidates for each sequence. The taxonomy of sequences without hits was determined using the RDP classifier with a minimum support threshold of 80% (39). In addition, we constructed a species-level table from the ASV table by clustering the ASVs assigned to the same reference sequence in eHOMD. ASVs without hits were excluded from the species-level analysis.

### Statistical analysis

All statistical analyses were performed using R software. The alpha diversity of the tongue microbiota in each group was compared using the Steel–Dwass test. The dissimilarity in bacterial composition between samples was evaluated using the Aitchison distance, based on a centered log-ratio (CLR) transformed abundance data at species level (40). The distances between the 18 months–4 months pairs and 18 months–mother pairs were compared using the Wilcoxon signed-rank test. In this study, we defined ASVs that were detected in 4-month infants but not in their mothers and/or more frequently detected in 4-month infants compared to their mothers (Fisher’s exact test *P* < 0.05) as 4-month specific ASVs. Furthermore, based on the distribution of the total abundance of 4-month specific ASVs, tongue microbiota profiles at 18 months of age with ≥30% or <30% of the total relative abundance of 4-month specific ASVs were defined as infant and mature profiles, respectively. For a hierarchical clustering approach, we constructed a modified species-level abundance table by dividing each species into two sub-units composed of 4-month specific ASVs and other ASVs. The hierarchical clustering approach for the mature profile was performed based on the Aitchison distances. A heatmap was generated using the *ComplexHeatmap* package in R (41). Differentially abundant species between the *S. salivarius*- and *Neisseria*-dominant profiles were identified using MaAsLin 2 (Microbiome Multivariable Associations with Linear Models) based on the CLR-transformed species-level table (42). To evaluate statistical differences in tongue microbiota profiles and clinical variables, Fisher’s exact test and Mann–Whitney *U* test were performed for categorical and continuous variables, respectively. Furthermore, a multivariate logistic regression analysis including variables with *P* < 0.05 in bivariate analysis was also performed. Linear trends in the proportions of the *S. salivarius*-dominant profile across frequencies of dietary intake were tested using the Cochran-Armitage trend test.

## Data Availability

The sequence data have been deposited in the NCBI/ENA/DDBJ Sequence Read Archive under the accession number DRA016799. The data presented in this study are available from the corresponding authors upon reasonable request.

## SUPPLEMENTAL MATERIAL

Fig. S1 mbio.01337-23-s0001.tif Histogram of total relative abundance of 4-month specific ASVs in tongue microbiota at 18 months of age.

Fig. S2 mbio.01337-23-s0002.tif Detection rate of 4-month specific ASVs in each group. Detection rate was calculated based on the rarefied (1,000 reads) ASV table.

Table S1 mbio.01337-23-s0003.docx Characteristics of infants at 18-month checkups.

Table S2 mbio.01337-23-s0004.docx Adjusted odds ratio for the *S. salivarius*-dominant profile at 18 months of age.

Table S3 mbio.01337-23-s0005.docx Characteristics at the 4-month checkup in infants with infant profile (IP), *S. salivarius*-profile (SSP), and *Neisseria*-dominant profile (NP) at the 18-month checkup.

Table S4 mbio.01337-23-s0006.docx Characteristics of infants with mature and infant profiles at the 18-month checkup after excluding antibiotic users.

Table S5 mbio.01337-23-s0007.docx Characteristics of infants with *S. salivarius*- and *Neisseria*-dominant profiles at the 18-month checkup based on clustering analysis after excluding antibiotic users.

Table S6 mbio.01337-23-s0008.docx Comparison between included and excluded subjects at the 4-month checkup.

ASM does not own the copyrights to Supplemental Material that may be linked to, or accessed through, an article. The authors have granted ASM a non-exclusive, world-wide license to publish the Supplemental Material files. Please contact the corresponding author directly for reuse.
