# Supplementary figures and images for: Establishment of tongue microbiota by 18 months of age and determinants of its microbial profile
Source: mBio. 2023 Oct 11;14(5):e01337-23. doi: 10.1128/mbio.01337-23 (PMC10653898; doi:10.1128/mbio.01337-23)

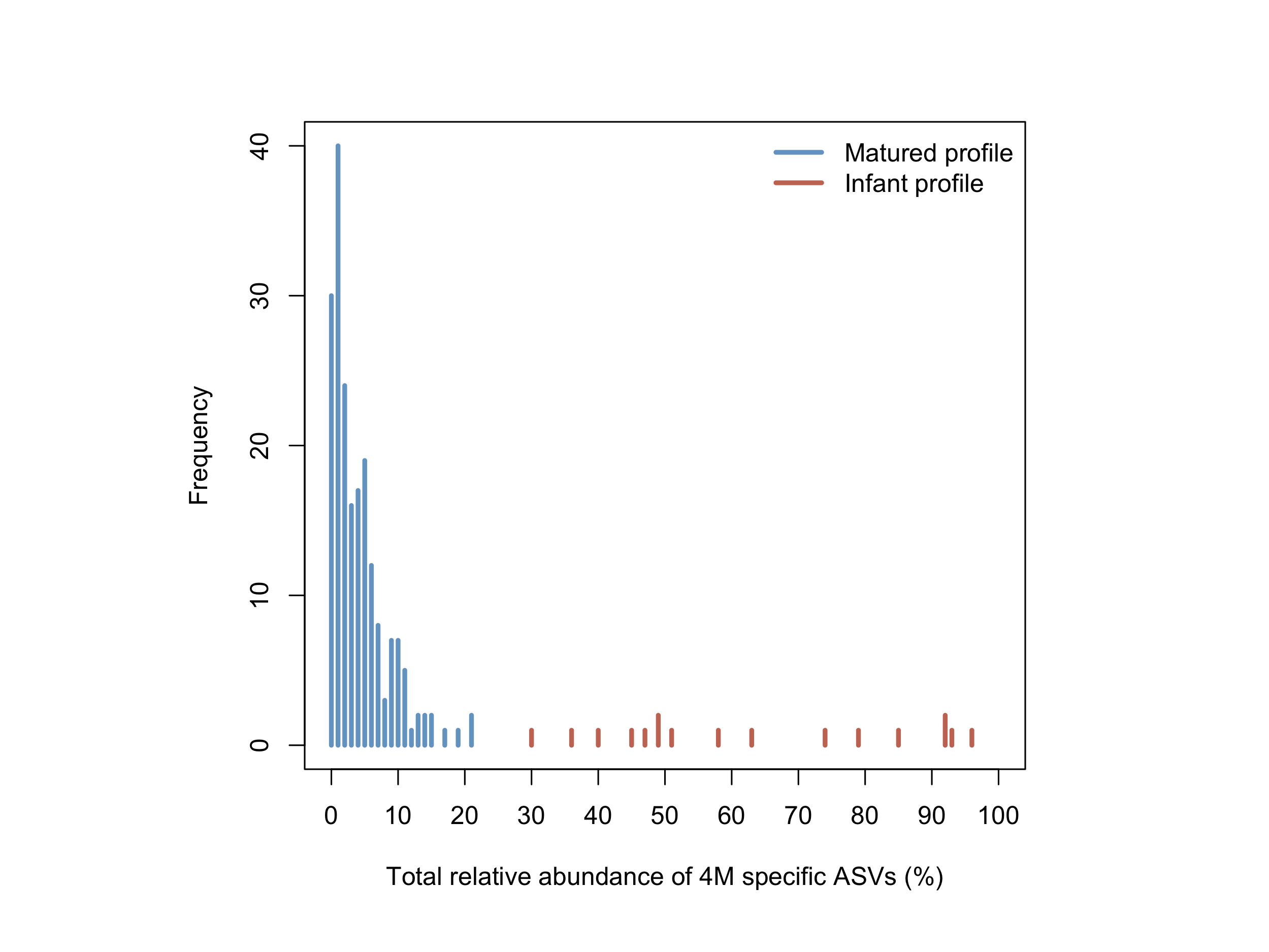

Supplement: Fig. S1 — Histogram of total relative abundance of 4-month specific ASVs in tongue microbiota at 18 months of age. [file mbio.01337-23-s0001.tif]

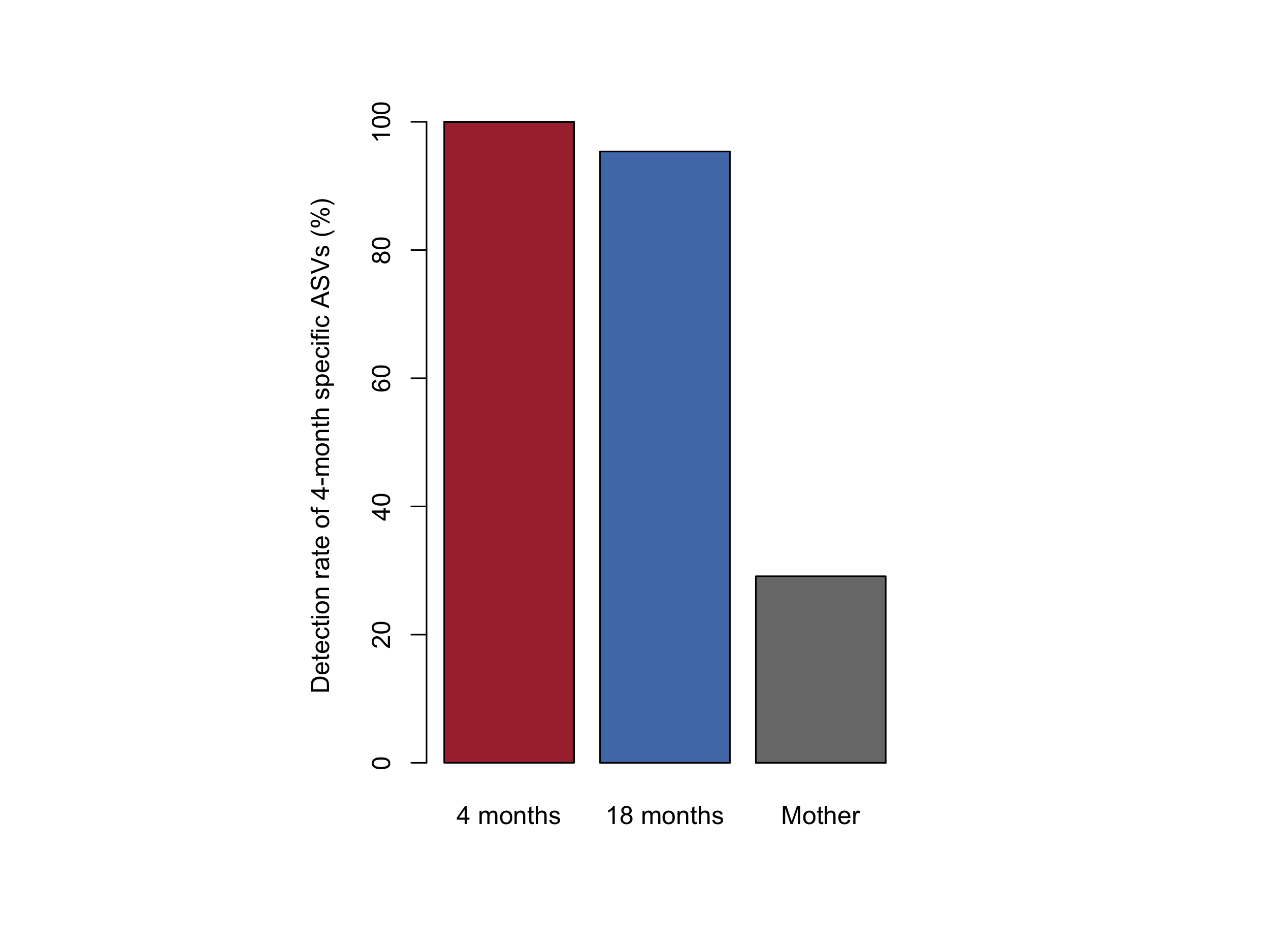

Supplement: Fig. S2 — Detection rate of 4-month specific ASVs in each group. Detection rate was calculated based on the rarefied (1,000 reads) ASV table. [file mbio.01337-23-s0002.tif]
