# Supplementary material for: Establishment of tongue microbiota by 18 months of age and determinants of its microbial profile
Source: mBio. 2023 Oct 11;14(5):e01337-23. doi: 10.1128/mbio.01337-23 (PMC10653898; doi:10.1128/mbio.01337-23)
Supplement: Table S1 — Characteristics of infants at 18-month checkups. [file mbio.01337-23-s0003.docx]

**Table S1. ﻿Characteristics of infants at 18-month checkups.**

|  | Infants (n=216) |
| --- | --- |
| Age (months) | 18.0 (17.6-18.5) |
| Sex |  |
| Boys | 105 (48.6) |
| Girls | 111 (51.4) |
| Feeding method |  |
| Breastfed | 42 (19.4) |
| Mixed-fed | 7 (3.2) |
| Formula-fed | 23 (10.6) |
| Weaned | 144 (66.7) |
| Number of present teeth | 16 (14-16) |
| Dental caries or white spot lesion |  |
| Detected | 3 (1.4) |
| Not detected | 209 (96.8) |
| Dental plaque accumulation |  |
| Considerable | 7 (3.2) |
| None or little | 205 (94.9) |
| Toothpaste with fluoride |  |
| Use | 130 (60.2) |
| Not use | 86 (39.8) |
| Fluoride treatment at dental office |  |
| Experienced | 51 (23.6) |
| Never | 164 (75.9) |
| Brushing of teeth by mother |  |
| Yes | 212 (98.1) |
| No | 4 (1.9) |
| Sharing tableware with adult |  |
| Sometimes | 105 (48.6) |
| Never | 111 (51.4) |
| Daycare center attendance |  |
| Yes | 119 (55.1) |
| No | 97 (44.9) |
| Antibiotic within a month |  |
| Use | 35 (16.2) |
| Not use | 180 (83.3) |
| Dietary intake (≥4 times per week) |  |
| Fruits | 158 (73.1) |
| Dairy products | 168 (77.8) |
| Sweetened beverages | 77 (35.6) |
| Sweet snacks | 129 (59.7) |

Data are presented as median values (interquartile range) for age and number of teeth present and n (%) for categorical variables.
