# Supplementary material for: Establishment of tongue microbiota by 18 months of age and determinants of its microbial profile
Source: mBio. 2023 Oct 11;14(5):e01337-23. doi: 10.1128/mbio.01337-23 (PMC10653898; doi:10.1128/mbio.01337-23)
Supplement: Table S2 — Adjusted odds ratio for the S. salivarius-dominant profile at 18 months of age. [file mbio.01337-23-s0004.docx]

**Table S2. Adjusted odds ratio for the *S. salivarius*-dominant profile at 18 months of age.**

|  | ﻿Adjusted odds ratio (95% CI) | P value |
| --- | --- | --- |
| Feeding method (ref. weaned) |  |  |
| Breastfed | 5.29 (2 - 15.6) | 0.001 |
| Mixed-fed | 3.18 (0.4 - 27.2) | 0.245 |
| Formula-fed | 3.14 (1.2 - 9.2) | 0.029 |
| Tableware sharing with adults (ref. never) | 2.82 (1.5 - 5.3) | 0.001 |
| Dietary intake ≥4 times per week (ref. <4 times) |  |  |
| Fruits | 0.65 (0.3 - 1.4) | 0.248 |
| Sweetened beverages | 2.12 (1 - 4.4) | 0.040 |
| Sweet snacks | 1.28 (0.6 - 2.6) | 0.495 |

Adjusted odds ratios were calculated using a multivariate logistic regression analysis. The model includes variables with P <0.05 in the bivariate analysis (Table 2). CI: confidence interval.
