# Supplementary material for: Establishment of tongue microbiota by 18 months of age and determinants of its microbial profile
Source: mBio. 2023 Oct 11;14(5):e01337-23. doi: 10.1128/mbio.01337-23 (PMC10653898; doi:10.1128/mbio.01337-23)
Supplement: Table S3 — Characteristics at the 4-month checkup in infants with infant profile (IP), S. salivarius-profile (SSP), and Neisseria-dominant profile (NP) at the 18-month checkup. [file mbio.01337-23-s0005.docx]

**Table S3. ﻿Characteristics at the 4-month checkup in infants with infant profile (IP), *S. salivarius*-profile (SSP), and *Neisseria*-dominant profile (NP) at the 18-month checkup.**

|  | Mature profile (MP) | | IP (n=17) | P value  (MP and IP) | P value  (SSP and NP) |
| --- | --- | --- | --- | --- | --- |
|  | SSP (n=100) | NP (n=99) |  |  |  |
| Boys | 52 (52) | 44 (44.4) | 9 (52.9) | 0.803 | 0.322 |
| Feeding method |  |  |  |  |  |
| Breastfeeding | 60 (60.6) | 50 (50.5) | 10 (58.8) | 0.874 | 0.335 |
| Mixed-feeding | 30 (30.3) | 35 (35.4) | 6 (35.3) |  |  |
| Formula-feeding | 9 (9.1) | 14 (14.1) | 1 (5.9) |  |  |
| Caesarean-section | 17 (17) | 24 (24.2) | 2 (11.8) | 0.534 | 0.224 |
| Antibiotic within a month | 5 (5) | 0 (0) | 1 (5.9) | 0.392 | 0.059 |
| ﻿Early delivery (<37 weeks) | 5 (5.1) | 6 (6.1) | 1 (5.9) | 1 | 1 |
| ﻿Low birth weight (<2,500 g) | 7 (7) | 15 (15.2) | 1 (5.9) | 1 | 0.074 |
| Current weight |  |  |  |  |  |
| Low (﻿﻿below -1 SD, <5,920.3 g) | 12 (12) | 25 (25.5) | 0 (0) | 0.095 | 0.048 |
| Mid (within ±1 SD) | 74 (74) | 62 (63.3) | 14 (82.4) |  |  |
| High ﻿(above +1 SD, >7,549.1 g) | 14 (14) | 11 (11.2) | 3 (17.6) |  |  |
| Kaup index (﻿g/cm^2^×10) |  |  |  |  |  |
| <16 | 21 (21) | 30 (30.6) | 0 (0) | 0.026 | 0.301 |
| ≥16 and <18 | 55 (55) | 48 (49) | 11 (64.7) |  |  |
| ≥18 | 24 (24) | 20 (20.4) | 6 (35.3) |  |  |

The frequencies were tested for differences between MP and IP or between SSP and NP using the Fisher’s exact test.
