# Supplementary material for: Establishment of tongue microbiota by 18 months of age and determinants of its microbial profile
Source: mBio. 2023 Oct 11;14(5):e01337-23. doi: 10.1128/mbio.01337-23 (PMC10653898; doi:10.1128/mbio.01337-23)
Supplement: Table S4 — Characteristics of infants with mature and infant profiles at the 18-month checkup after excluding antibiotic users. [file mbio.01337-23-s0006.docx]

**Table S4. ﻿Characteristics of infants with mature and infant profiles at the 18-month checkup after excluding antibiotic users.**

|  | Mature profile  (n=166) | Infant profile  (n=14) | P value |
| --- | --- | --- | --- |
| Age (months) | 18.0 (17.5-18.5) | 17.9 (17.7-18.1) | 0.405 |
| Boys | 73 (44.0) | 7 (50.0) | 0.782 |
| Feeding method |  |  |  |
| Breastfed | 24 (14.5) | 12 (85.7) | <0.001 |
| Mixed-fed | 4 (2.4) | 1 (7.1) |  |
| Formula-fed | 20 (12) | 1 (7.1) |  |
| Weaned | 118 (71.1) | 0 (0) |  |
| Number of present teeth | 16 (14-16) | 15.5 (13.25-16) | 0.949 |
| Dental caries or white spot lesion | 0 (0) | 2 (14.3) | 0.006 |
| Dental plaque accumulation | 2 (1.2) | 4 (28.6) | <0.001 |
| Toothpaste with fluoride | 103 (62.0) | 6 (42.9) | 0.168 |
| Fluoride treatment at dental office | 43 (25.9) | 2 (14.3) | 0.523 |
| Brushing of teeth by mother | 163 (98.2) | 13 (92.9) | 0.279 |
| Tableware sharing with adults | 75 (45.2) | 7 (50.0) | 0.785 |
| Daycare center attendance | 83 (50.0) | 6 (42.9) | 0.782 |
| Dietary intake (≥4 times per week) |  |  |  |
| Fruits | 123 (74.1) | 9 (64.3) | 0.529 |
| Dairy products | 129 (78.2) | 9 (64.3) | 0.317 |
| Sweetened beverages | 57 (34.3) | 4 (28.6) | 0.775 |
| Sweet snacks | 98 (59.0) | 11 (78.6) | 0.254 |

Data are presented as median values (interquartile range) for age and number of teeth present and n (%) for categorical variables.
