# Supplementary material for: Establishment of tongue microbiota by 18 months of age and determinants of its microbial profile
Source: mBio. 2023 Oct 11;14(5):e01337-23. doi: 10.1128/mbio.01337-23 (PMC10653898; doi:10.1128/mbio.01337-23)
Supplement: Table S5 — Characteristics of infants with S. salivarius- and Neisseria-dominant profiles at the 18-month checkup based on clustering analysis after excluding antibiotic users. [file mbio.01337-23-s0007.docx]

**Table S5. ﻿Characteristics of infants with *S. salivarius*- and *Neisseria*-dominant profiles at the 18-month checkup based on clustering analysis after excluding antibiotic users.**

|  | *S. salivarius*-dominant  profile (n=77) | *Neisseria*-dominant  profile (n=89) | P value |
| --- | --- | --- | --- |
| Age (months) | 18.0 (17.5-18.6) | 18.0 (17.6-18.5) | 0.644 |
| Boys | 36 (46.8) | 37 (41.6) | 0.533 |
| Feeding method |  |  |  |
| Breastfed | 15 (19.5) | 9 (10.1) | 0.016 |
| Mixed-fed | 2 (2.6) | 2 (2.2) |  |
| Formula-fed | 14 (18.2) | 6 (6.7) |  |
| Weaned | 46 (59.7) | 72 (80.9) |  |
| Number of present teeth | 16 (14-16) | 16 (14-16) | 0.333 |
| Dental plaque accumulation | 1 (1.3) | 1 (1.1) | 1 |
| Toothpaste with fluoride | 53 (68.8) | 50 (56.2) | 0.11 |
| Fluoride treatment at dental office | 18 (23.4) | 25 (28.1) | 0.595 |
| Brushing of teeth by mother | 75 (97.4) | 88 (98.9) | 0.597 |
| Tableware sharing with adults | 46 (59.7) | 29 (32.6) | <0.001 |
| Daycare center attendance | 38 (49.4) | 45 (50.6) | 1 |
| Dietary intake (≥4 times per week) |  |  |  |
| Fruits | 51 (66.2) | 72 (80.9) | 0.035 |
| Dairy products | 57 (75) | 72 (80.9) | 0.45 |
| Sweetened beverages | 33 (42.9) | 24 (27) | 0.035 |
| Sweet snacks | 51 (66.2) | 47 (52.8) | 0.085 |

Data are presented as median values (interquartile range) for age and number of teeth present and n (%) for categorical variables.
