# Supplementary material for: Establishment of tongue microbiota by 18 months of age and determinants of its microbial profile
Source: mBio. 2023 Oct 11;14(5):e01337-23. doi: 10.1128/mbio.01337-23 (PMC10653898; doi:10.1128/mbio.01337-23)
Supplement: Table S6 — Comparison between included and excluded subjects at the 4-month checkup. [file mbio.01337-23-s0008.docx]

**Table S6. ﻿Comparison between included and excluded subjects at the 4-month checkup.**

|  | Included (n=216) | Excluded (n=232) | P value |
| --- | --- | --- | --- |
| Boys | 105 (48.6) | 112 (48.3) | 1 |
| Feeding method |  |  |  |
| Breastfeeding | 120 (55.8) | 135 (58.4) | 0.17 |
| Mixed-feeding | 71 (33) | 60 (26) |  |
| Formula-feeding | 24 (11.2) | 36 (15.6) |  |
| Caesarean-section | 43 (19.9) | 45 (19.4) | 0.906 |
| Antibiotic within a month | 6 (2.8) | 14 (6) | 0.112 |
| ﻿Early delivery (<37 weeks) | 12 (5.6) | 9 (3.9) | 0.503 |
| ﻿Low birth weight (<2,500 g) | 23 (10.6) | 14 (6) | 0.087 |
| Current weight |  |  |  |
| Low (﻿﻿below -1 SD, <5,920.3 g) | 37 (17.2) | 25 (10.9) | 0.089 |
| Mid (within ±1 SD) | 150 (69.8) | 164 (71.3) |  |
| High ﻿(above +1 SD, >7,549.1 g) | 28 (13) | 41 (17.8) |  |
| Kaup index (﻿g/cm^2^×10) |  |  |  |
| <16 | 51 (23.7) | 50 (21.7) | 0.866 |
| ≥16 and <18 | 114 (53) | 127 (55.2) |  |
| ≥18 | 50 (23.3) | 53 (23) |  |

Frequencies were tested for differences between the included and excluded participants using Fisher’s exact test.
